# Implementation and evaluation of an optimized surgical clerkship teaching model utilizing ChatGPT

**DOI:** 10.1186/s12909-024-06575-9

**Published:** 2024-12-27

**Authors:** Yi Huang, Bei-bei Xu, Xiu-yan Wang, Yun-cheng Luo, Miao-miao Teng, Xuejian Weng

**Affiliations:** 1 Department of General Surgery, Wenzhou Third Clinical Institute Affiliated to Wenzhou Medical University, The Third Affiliated Hospital of Shanghai University, Wenzhou People’s Hospital, Wenzhou Maternal and Child Health Care Hospital, Wenzhou, Zhejiang 325000 China; 2 Department of Gastroenterology, Wenzhou Third Clinical Institute Affiliated to Wenzhou Medical University, The Third Affiliated Hospital of Shanghai University, Wenzhou People’s Hospital, Wenzhou Maternal and Child Health Care Hospital, No. 57 Cang Hou Street, Wenzhou, Zhejiang 325000 China; 3 Department of Gastroenterology, Postgraduate Training Base Alliance of Wenzhou Medical University, Wenzhou, Zhejiang 325000 China

**Keywords:** Artificial intelligence, ChatGPT, Compliance, Learning effectiveness, Medical education, Satisfaction

## Abstract

**Objective:**

This study aims to explore the effect of an innovative teaching model incorporating ChatGPT on medical students’ learning outcomes, compliance with learning activities, and overall satisfaction with the learning process.

**Methods:**

A cohort of 64 students participating in general surgery clerkships at Wenzhou People’s Hospital during the 2022–2023 academic year were randomly assigned into 4 groups, each comprising 16 students. Two of these groups were designated as the study group, where ChatGPT was employed as a supplementary educational tool. The remaining 2 groups served as control groups and used traditional multimedia-based learning methods. Outcomes, including learning effectiveness, compliance, and satisfaction, were evaluated using questionnaires and tests.

**Results:**

The study groups exhibited significantly higher levels of compliance and satisfaction compared to the control groups. Specifically, the study groups exhibited significantly greater compliance in both pre-class preparation and post-class review activities (*P* < 0.05). During classroom teaching, Group 1 of the study group achieved significantly higher compliance than the control groups (*P* < 0.0001), while Group 2 of the study group showed significantly higher compliance than Group 1 (*P* < 0.001). In terms of seeking feedback and assistance, both Groups 1 and 2 of the study group had significantly higher compliance compared to Group 1 of the control group (*P* < 0.01, *P* < 0.001 respectively). Overall satisfaction was significantly higher in the study groups compared to the control groups (*P* < 0.0001), particularly with respect to course organization (*P* < 0.001, *P* < 0.05).

**Conclusion:**

The incorporation of ChatGPT into the surgical clerkship teaching model substantially enhances learner compliance and satisfaction, offering notable advantages in educational effectiveness.

## Background

ChatGPT, formally known as Chat Generative Pre-trained Transformer (ChatGPT), is a natural language processing tool built on deep learning technology, initially launched on November 30, 2022 [1]. It is specifically designed for dialogue applications capable of generating natural and coherent conversations [2]. In the field of medical education, ChatGPT has demonstrated considerable potential as a teaching tool [3]. A recent study revealed that ChatGPT achieved a performance level comparable to third-year medical students in the United States Medical Licensing Examination (USMLE) [4]. Moreover, ChatGPT’s ability to provide logical responses while maintaining contextual relevance indicates its potential as a virtual medical tutor.

Given its promising advantages in medical education, educators can leverage this advanced tool to mentor and nurture future physicians. In this study, we incorporated ChatGPT into clinical clerkship supervision by employing it to simulate patient interactions, clinical scenarios, and case situations. This approach provided medical students with opportunities to practice collecting medical histories, engaging in case discussions, and making clinical decisions. The objectives were to supplement instructor teaching skills, accelerate instructors’ growth path, reduce teaching pressure, stimulate student interest, and enhance overall learning efficiency.

## Methods

All model tests were conducted using the ChatGPT version released on December 15, 2022. Questions were manually entered into the ChatGPT interface and the resulting responses were directly copied into a shared electronic document for review (Appendix 1).

### Personnel selection

This study involved two classes of students who were on clerkship at the General Surgery Department of Wenzhou People’s Hospital during the 2022–2023 academic year, all from Wenzhou Medical University. A total of 64 students were randomly assigned into 4 groups, with 16 students in each group. In each class, 2 groups were randomly assigned to the same instructor but received different teaching methods for the course “Intestinal Obstruction.” One group received AI-assisted teaching, while the other was taught using traditional methods. The control group employed a conventional multimedia teaching model, incorporating clinical cases with two-dimensional text, images, and videos. In contrast, the study group utilized ChatGPT to assist with simulated patients, clinical scenarios, and case discussions, facilitating engagement in clinical decision-making. Before class, we will divide students in different classes into the control group. Before class, we will send PPT and other materials of the teaching course to the group, which do not involve any suggestion of using AI. After class, we will design a questionnaire to exclude the data of students who use AI methods independently from the final analysis. In this experiment, none of the students in the control group used AI independently. ChatGPT group1 and reference group 1 were from the same large class, and chatGPT group 2 and reference group 2 were from the same large class. We randomly sorted the students in the same large class and randomly divided them into the control group and the research group. It can be considered that there was no difference between the two chatGPT groups and the two control groups. In addition, statistical analysis of the subsequent results will also be conducted between the chatGPT group and between the two control groups.

### Specific exercise protocol

Control Group: The traditional teaching model was used in the department’s demonstration classroom, with instruction provided by a senior physician. The teaching protocol included the following components: ① Pre-class preparation: Relevant disease diagnosis and treatment guidelines were distributed to students a week before the teaching session. ② In-class instruction: Classic multimedia methods were used to present clinical cases, covering topics such as epidemiology, etiology, clinical manifestations, diagnosis, treatment, and prognosis of intestinal obstruction. This included the use of images to illustrate basic imaging findings and interpretations based on the latest guidelines. Post-class review: Students were required to review the content covered in class and apply the Fermi-style learning method to enhance their understanding of the disease [5]. They were also tasked with completing post-class assessment exercises.

Study Group: The innovative teaching mode incorporating ChatGPT was implemented in the department’s demonstration classroom, with instruction provided by a senior physician. The specific teaching methods included: ① Pre-class preparation: Teachers introduced the ChatGPT operation guidelines in a group chat, guiding students to familiarize themselves with its use. ② In-class instruction: Students engaged in diagnosing and treating “virtual patients” with guidance from the teaching physician, receiving positive feedback on their treatment decisions when they were correct. Following the refinement of course content, ChatGPT was utilized to administer tests, provide access to the latest literature, and verify the accurate extraction of literature abstracts. ③ Post-class review: Students were required to review the content using ChatGPT in an “I ask ChatGPT, and it provides answers” format, similar to the Feynman Technique. They also completed post-class assessment exercises.

### Evaluation system

The training effects of the study group and the control group were quantitatively evaluated and assessed across three dimensions: learning effectiveness, learning compliance, and learning satisfaction. The evaluation system was structured as follows:

1. Learning Effects: A standardized assessment test was developed, encompassing the key content to be mastered, specified in the teaching syllabus. The training effect was evaluated using uniform criteria to ensure consistency in measuring learning outcomes.
2. Learning Compliance. A compliance statistics table was prepared, encompassing 4 components: the development of a learning plan, autonomous pre-class preview and post-class review, participation in classroom teaching, and seeking feedback and assistance. The learning compliance of the 4 student groups was evaluated and analyzed statistically. The proportions of complete compliance (100%), partial compliance (80%), basic compliance (60%), and non-compliance (< 60%) were calculated for each group, and intergroup comparisons were conducted to evaluate differences in compliance levels.

Full compliance (100%): Students strictly follow the study plan or recommendations without deviation. Represents students’ high attention to learning requirements and execution, and can fully achieve learning goals.

Relative adherence (80%): Students follow a study plan or recommendation most of the time, with occasional small deviations or flexible adjustments. Indicates that students have a high degree of compliance with the learning plan and can effectively complete most of the learning tasks, but may need to improve in some aspects.

Basic compliance (60%): Students sometimes follow study plans or recommendations, but with more deviation or flexibility.

Indicates that the student’s implementation of the study plan is not consistent enough and may require further guidance and support to improve compliance.

Non-adherence (less than 60%): Students rarely or hardly follow study plans or recommendations and often deviate from stated goals. This may indicate that the student is experiencing difficulties or lack of motivation in the learning process and needs extra help and motivation to improve study habits and attitudes.

(3) Learning Satisfaction. A satisfaction survey was developed and conducted to evaluate the training experiences of the 4 student groups. The survey comprised 4 key areas: Satisfaction with course content: This included ease of understanding and mastery of the course content, the guidance and insight provided by the textbook, and the relevance of typical cases. Satisfaction with the faculty: This encompassed communication skills, familiarity with course content, and clarity and precision in analyzing and responding to questions. Satisfaction with teaching methods: This assessed the design and analytical thinking applied to course cases. Satisfaction with course organization and arrangement: This involved evaluating the effectiveness of guiding interactive classroom discussions and the rational allocation of time for key content. The total survey score was 100 points, with each item scored on a hierarchical system, where the highest score for any single item was 25 points.

### Statistical analysis

Statistical analysis was conducted using GraphPad Prism 10.1.2. Measurement data are expressed as mean ± standard deviation (x ± s). For intergroup comparisons, analysis of variance (ANOVA) was employed if the data satisfied the conditions for parametric testing; otherwise, the rank-sum test was used. Categorical data are reported as frequencies and percentages, with intergroup comparisons conducted using the chi-squared test (χ² test). Pairwise comparisons between groups were adjusted using the Bonferroni correction for the significance level. A significance threshold of *P* < 0.05 was established.

## Results

### Learning effects

**Fig. 1:**
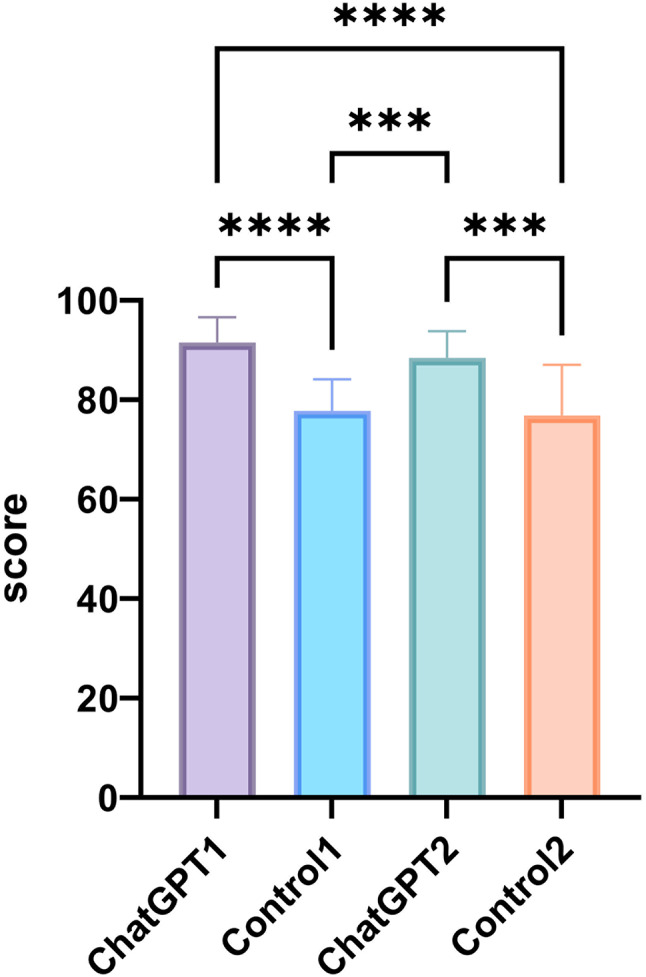
Theoretical knowledge test scores of students in the two groups. Differences between groups are indicated by *P < 0.05, **P < 0.01, ***P < 0.001, ****P < 0.0001

The theoretical knowledge assessment scores for Study Group 1 were significantly higher compared to Control Group 1 and Control Group 2 (*P* < 0.0001 for both comparisons as shown in Fig. 1. Similarly, Study Group 2 also achieved significantly higher scores than both Control Group 1 and Control Group 2 (*P* < 0.001 for both comparisons). No significant differences were observed in theoretical knowledge assessment scores between Study Groups 1 and 2, nor between Control Groups 1 and 2.

### Learning compliance

**Fig. 2:**
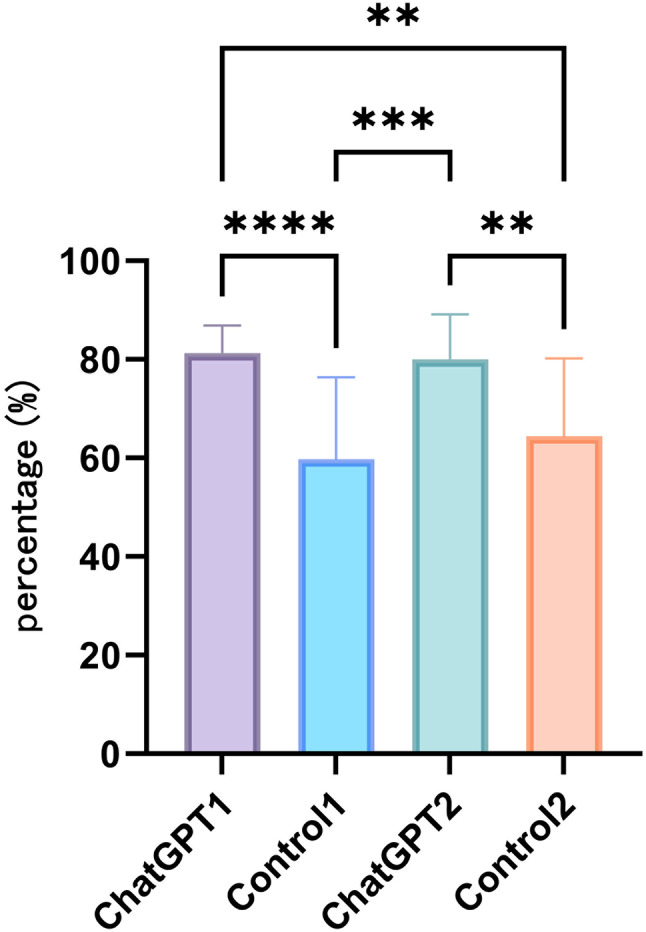
Total compliance percentage of students in the four groups. Differences among groups are indicated by *P < 0.05, **P < 0.01, ***P < 0.001, ****P < 0.0001

Average compliance score is in Table 1. The total compliance scores as illustrated in Fig. 2, indicated that Study Group 1 exhibited significantly higher compliance compared to Control Group 1 and Control Group 2 (*P* < 0.0001, *P* < 0.01 respectively). Similarly, Study Group 2 demonstrated significantly higher compliance than Control Group 1 and Control Group 2 (*P* < 0.001, *P* < 0.01 respectively).

**Table 1:** Average score of compliance

	Total score of compliance	Generate study plan	Pre-class preview & after-class review	Participate in classroom teaching	Seek for help and feeedback
ChatGPT group 1	81.25	18.75	21.25	22.19	19.06
Control group 1	59.69	15.94	16.25	13.75	13.75
ChatGPT group 2	80.00	18.13	20.94	21.25	19.69
Control group 2	64.38	15.94	15.63	16.88	15.94

**Fig. 3:**
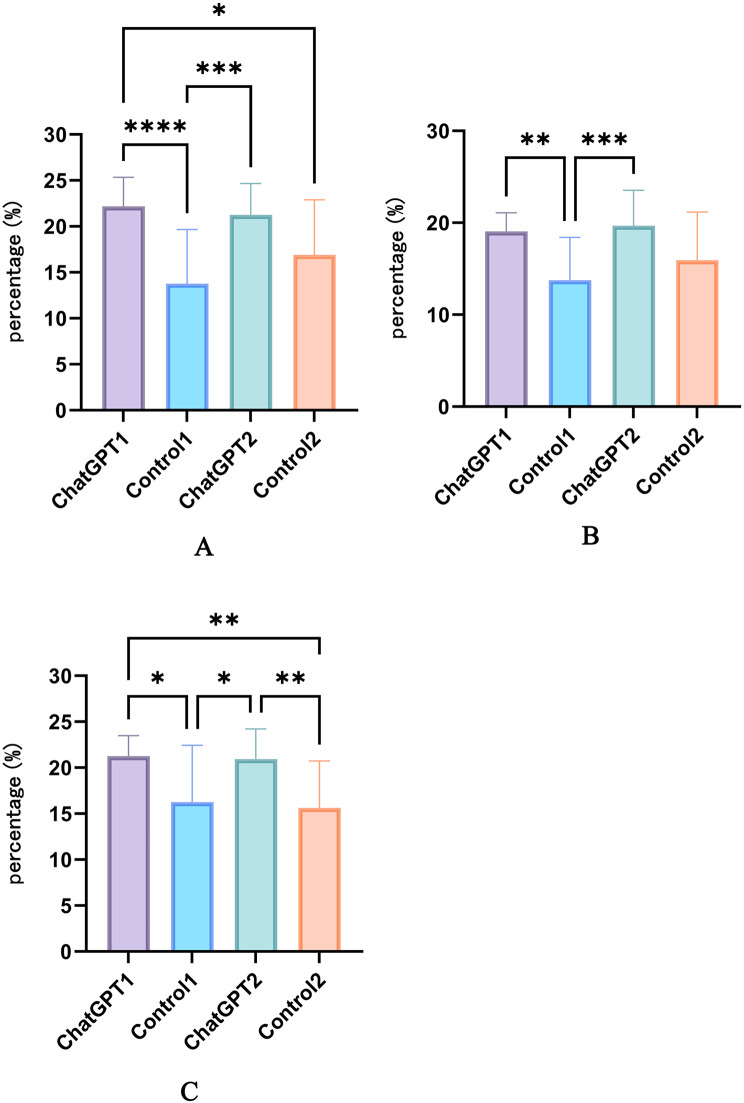
(**A**) Compliance of students in the four groups with classroom teaching participation. (**B**) Compliance of students in the four groups with seeking feedback and help after class. (**C**) Compliance of students in the four groups with autonomous pre-class preparation and post-class review. Differences among groups are indicated by *P < 0.05, **P < 0.01, ***P < 0.001, ****P < 0.0001

Regarding the development of a learning plan, no significant differences were observed among the groups. However, in terms of participating in classroom teaching (Fig. 3A), Study Group 1 demonstrated higher compliance compared to Control Group 1 and Control Group 2 (*P* < 0.0001, *P* < 0.05 respectively). Study Group 2 also showed higher compliance than Control Group 1 (*P* < 0.001). No significant differences in compliance were observed between Study Groups 1 and 2, nor between Control Groups 1 and 2.

In seeking feedback and assistance (Fig. 3B), both Study Group 1 and Study Group 2 exhibited higher compliance compared to Control Group 1 (*P* < 0.01, *P* < 0.001, respectively), with no significant differences noted among the other groups. For pre-class independent preview and post-class independent review (Fig. 3C), Study Group 1 showed higher compliance than Control Group 1 and Control Group 2 (*P* < 0.05, *P* < 0.01, respectively). Similarly, Study Group 2 also demonstrated higher compliance compared to Control Group 1 and Control Group 2 (*P* < 0.05, *P* < 0.01, respectively). No significant differences were observed in compliance between Study Groups 1 and 2, nor between Control Groups 1 and 2 in this aspect.

### Learning satisfaction

**Fig. 4:**
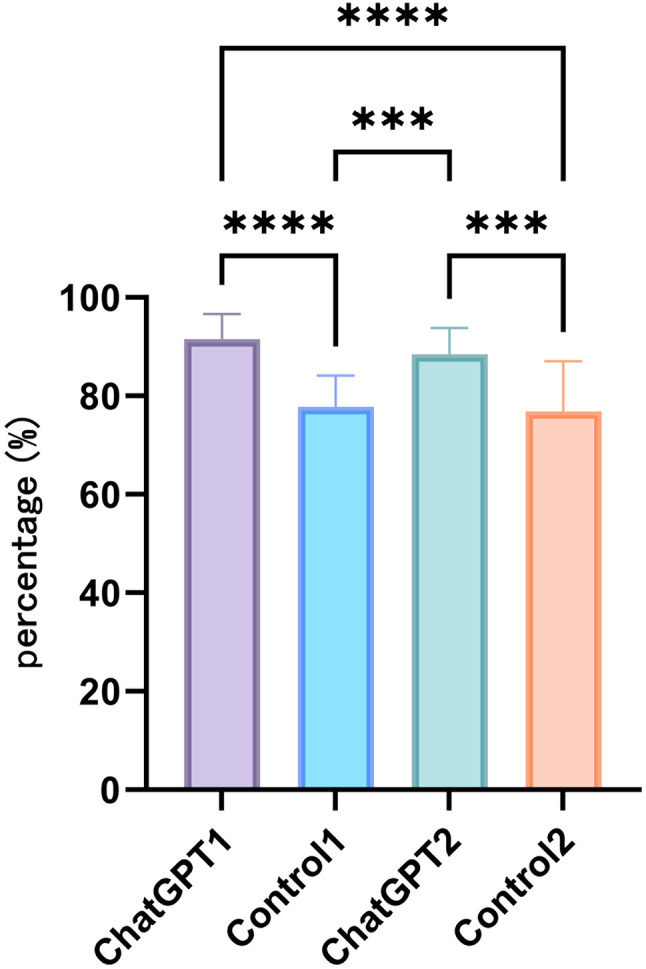
Total satisfaction percentage of students in the four groups. Differences among groups are indicated by *P < 0.05, **P < 0.01, ***P < 0.001, ****P < 0.0001

Based on the total satisfaction scores (Fig. 4), Study Group 1 reported higher satisfaction scores compared to Control Group 1 and Control Group 2 (*P* < 0.0001 for both comparisons). Similarly, Study Group 2 also showed higher satisfaction scores than Control Group 1 and Control Group 2 (*P* < 0.001 for both comparisons). No significant differences were observed in total satisfaction scores between Study Groups 1 and 2, nor between Control Groups 1 and 2.

**Fig. 5:**
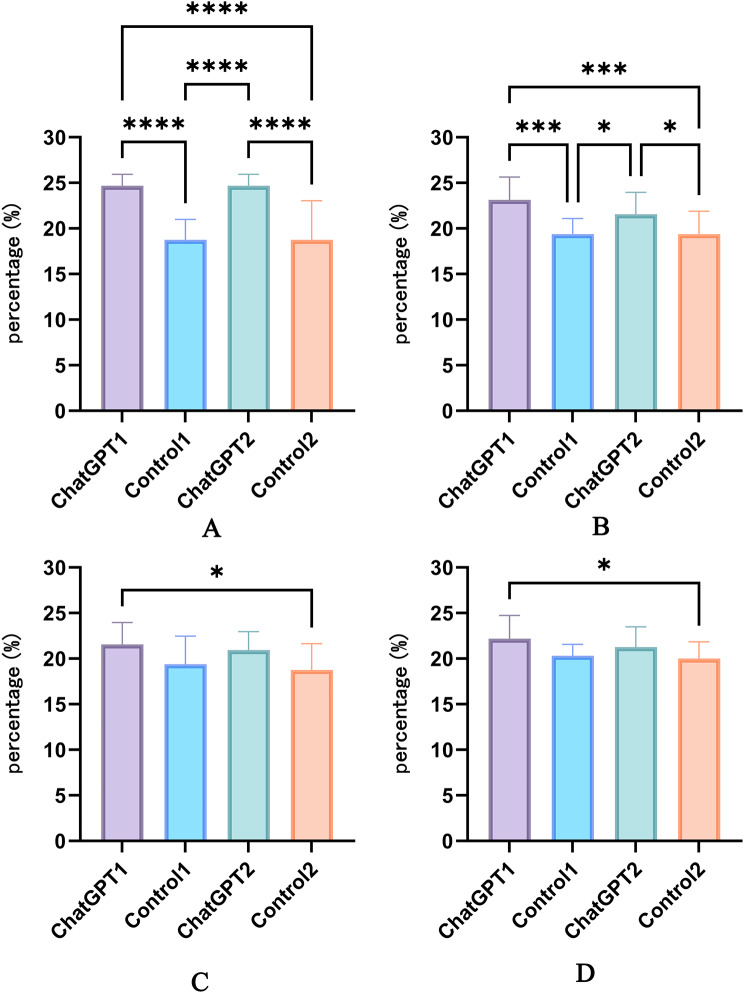
(**A**) Comparison of teaching method satisfaction among students in the four groups. (**B**) Comparison of course organization satisfaction among students in the four groups. (**C**) Comparison of course content satisfaction among students in the four groups. (**D**) Comparison of satisfaction with faculty strength among students in the four groups. Differences among groups are indicated by *P < 0.05, **P < 0.01, ***P < 0.001, ****P < 0.0001

In terms of satisfaction with teaching methods (Fig. 5A), both Study Groups 1 and 2 exhibited higher satisfaction scores compared to Control Groups 1 and 2 (*P* < 0.0001 for both comparisons). No significant differences were observed in satisfaction with teaching methods between Study Groups 1 and 2, nor between Control Groups 1 and 2 (*P* > 0.05).

In terms of satisfaction with course organization (Fig. 5B), Study Group 1 reported higher satisfaction scores compared to Control Group 1 and Control Group 2 (*P* < 0.001 for both comparisons). Similarly, Study Group 2 exhibited higher satisfaction scores than Control Group 1 and Control Group 2 (*P* < 0.05 for both comparisons). No significant differences were observed in satisfaction with course organization between Study Groups 1 and 2, nor between Control Groups 1 and 2.

In terms of satisfaction with course content and instructors (Fig. 5C and D), only Study Group 1 reported higher satisfaction scores compared to Control Group 2 (*P* < 0.05). No significant differences were observed in satisfaction among the remaining groups.

## Discussion

Technological breakthroughs are significantly advancing medical education. Currently, medical curriculum development emphasizes the enhancement of teaching methods through medical simulation, literature discussions, and research. There is a growing advocacy for integrating artificial intelligence and clinical guidelines into teaching to better cultivate students’ clinical reasoning and logical thinking abilities.

Multiple studies have demonstrated the potential benefits of ChatGPT in medical education. Chatbots such as ChatGPT can be a powerful tool for improving health literacy, particularly among students and young learners [6]. Firstly, ChatGPT offers rapid and immediate access to extensive medical information, aiding novice medical students in analyzing complex medical data [7]. Secondly, by creating case studies and scenarios, ChatGPT helps students refine and enhance their diagnostic and treatment planning skills, thereby improving their clinical reasoning abilities and preparedness for real-world clinical situations [8]. Thirdly, ChatGPT can support academic tasks by answering questions and drafting abstracts. Its ability to create outlines and literature reviews can streamline medical research. Additionally, it also facilitates the summarization of relevant publications and highlights important findings, aiding medical researchers in navigating the vast amount of material available online [9]. Finally, ChatGPT enables personalized learning for students by serving as a virtual tutor or assistant, assisting students with assignments and fostering interactive learning experiences [10]. 

In this study, ChatGPT was utilized in four key roles across the pre-class, in-class, and post-class phases of medical education. During the pre-class preparation phase, students were able to query ChatGPT about any problems they encountered, facilitating an initial understanding of fundamental medical concepts, terminology, and cases. In one study, a series of images could be generated from descriptive text using a deep learning model based on generative adversarial networks. The tool is used in the visual narrative process to facilitate technology-enhanced learning and improve logical reasoning skills [11]. Deep learning models based on generative adversarial networks play a key role in simulating various types of learning environments and help develop practical skills in virtual teaching assistant models. Experimental results show that this model improves students’ learning effect and enhances their learning motivation and learning ability [12]. In the classroom, ChatGPT was employed to simulate patient interactions, providing a platform for students to practice diagnostic and communication skills in a safe and controlled environment. In their interactions with ChatGPT, students are free to practice diagnostic and communication skills without the risks that a real patient might pose. A false diagnosis or miscommunication has no real impact on the patient, allowing students to learn from trial and error. ChatGPT is readily available and students can practice at their own learning pace and needs, without relying on a specific time and place. This flexibility makes learning more efficient and convenient. ChatGPT can simulate a variety of clinical scenarios and patient characteristics to provide a diverse interactive experience. Students are exposed to different conditions and patient backgrounds, thereby improving their ability to cope with complex situations. After class, students could engage with ChatGPT individually or in study groups, discussing practice questions provided by the tool, addressing difficult or challenging questions, and exploring the material from various perspectives. Throughout the interactive process, students continually assessed their understanding of the material, identified their weaknesses, and adjusted their learning strategies and focus areas in a timely manner to target specific areas for review and reinforcement, ensuring they remained on track [13]. Similarly, instructors could utilize ChatGPT to collect teaching resources and relevant cases studies during the lesson preparation phase. By leveraging ChatGPT, they could enhance student engagement in the classroom and use the tool after class to gather and analyze student feedback on the teaching process. Additionally, students could use ChatGPT to quickly resolve any confusion related to professional knowledge. With the training of the ChatGPT model, medical students and physicians can enhance their clinical reasoning and decision-making skills, thereby improving the performance of case analysis and diagnosis. In addition, ChatGPT provides medical learners with a personalized and efficient learning experience through simulated conversations, intelligent tutoring, and automated question and answer, thereby deepening students’ understanding of medical knowledge [14]. 

The results of this study indicate that the theoretical scores of the study groups were significantly higher than those of the control groups, reflecting better learning outcomes. No significant differences in scores were observed between the two study groups or between the two control groups. This suggests that the application of ChatGPT in the study groups resulted in a superior understanding and mastery of theoretical knowledge compared to the traditional teaching methods used in the control groups.

The teaching satisfaction results of this study indicate that students in the study groups, who utilized ChatGPT, reported significantly higher total satisfaction scores, as well as better ratings for course organization and teaching methods, compared to the control groups. The differences in satisfaction with course content and instructors were relatively minor, suggesting that the use of ChatGPT as a teaching aid, through its novel and engaging interactive question-and-answer format, strong interactivity, and structured approach, appears to enhance students’ engagement and involvement in learning. This indicates that ChatGPT can effectively foster greater interest and promote educational outcomes. The most notable difference between the teaching methods lies in classroom execution; ChatGPT’s capability to simulate various scenarios and conduct case analyses, combined with providing access to additional teaching resources, significantly improves medical students’ clinical application skills.

The study’s evaluation of learning compliance encompassed four aspects. The findings indicate that there were no significant differences among the groups in terms of establishing learning plans. However, for the other three aspects—autonomous pre-class preparation and post-class review, participation in classroom teaching, and seeking feedback and assistance—the study groups exhibited significantly higher ratings compared to the control groups. In many studies and statistical analyses, a “higher score” is often seen as a positive result, meaning that the study group performed better in something. The evaluation indicators of this study are all positive, and it can be considered that “higher score” indicates better performance of the research group, which is a positive result. This suggests that incorporating ChatGPT as a teaching aid enhances students’ learning compliance by promoting active learning, encouraging inquiry-based learning, and improving their interest and capacity for autonomous learning.

While compliance improvements are evident, continuous deepening of understanding before, during, and after class also contributes to enhanced logical thinking and analytical skills. Notably, the study found a relatively low rate of student questions and requests for help, during and after class. The observed differences between the study and control groups may be attributed to ChatGPT’s ability to help students overcome shyness and be non-judgmental about mistakes. The AI tool helps students overcome hesitations, enabling them to ask questions freely and repeatedly without fear of judgment or negative interactions. By generating learning materials based on each student’s learning state and needs, ChatGPT empowers students to take a more autonomous approach to learning and have an educational experience tailored to their preferences. Such interactions facilitate timely clarification, deeper understanding, and mastery of the material.

ChatGPT can also tailor individualized learning plans and materials for each student to accommodate varying learning styles and abilities within the classroom. This personalized approach fosters a positive feedback loop, enhancing students’ learning capabilities.

The application of ChatGPT in medical education remains a subject of considerable debate. While ChatGPT offers innovative functionalities and potential advantages, it also raises several ethical concerns and practical concerns, the potential for misuse, particularly in the realms of education and academia [15]. As a chatbot, ChatGPT lacks the ability to think critically like a human, limiting its capacity to interpret and analyze medical information beyond its programmed algorithms. It does not possess the judgment or discernment required for ethical or legal aspects of medical practice and may pose risks related to data breaches and privacy violations [16, 17]. 

The rise of AI tools like ChatGPT has led to academic dishonesty, with reports of students using the technology to cheat on essay assignments [18]. Some research suggests that ChatGPT may not be a reliable resource for complex issues that require advanced skills and knowledge [19]. Additionally, scholars have been concerned about ChatGPT’s reliability as a credible source of information [20]. According to many educationalists, ChatGPT can easily be used to cheat by students taking communication and philosophy courses, but is easy to identify. A growing concern is that students will eventually lose the ability to come up with original ideas and will not be able to present proper arguments to prove a point [21]. Technological accessibility is a challenge. Effective use of ChatGPT depends on network connectivity and device availability, which can be problematic in different regions and among specific student populations. Policies must be developed to use ChatGPT in different technical environments [22]. One concern is the potential devaluation of cooperative learning in medical education, particularly in traditional approaches such as PBL, CBL, and TBL. Collaboration and teamwork are crucial in these approaches, and ChatGPT may unintentionally reduce the importance of human-to-human interactions. Maintaining a balance between technology and relationships is essential for effective learning. While ChatGPT enhances PBL through personalized instruction, educators must emphasize the enduring importance of patient-based learning and teamwork. Despite ChatGPT’s simulation capabilities and theoretical insights, it cannot replace practical experience gained through real-world interactions, especially in medical education. Acknowledging the limitations of models is essential to prevent over-reliance on simulation learning. Seamlessly embedding ChatGPT into existing curricula is a challenge that requires educators to invest time in designing and integrating AI-driven components that align with the overall learning goals [23]. Given these considerations, it is essential to use ChatGPT judiciously as an auxiliary learning tool, complementing rather than replacing traditional educational methods and research techniques and be aware of ChatGPT’s limitations.

## Conclusion

In summary, our research indicates that ChatGPT offers substantial benefits in enhancing simulated patient interactions, clinical scenarios, and case-based learning. By facilitating case discussions and clinical decision-making, ChatGPT not only improved students’ learning outcomes but also enhanced their compliance and satisfaction with the educational process. This innovative teaching tool has proven effective in deepening students’ understanding of theoretical knowledge and enhancing their practical skills in clinical settings. These findings highlight ChatGPT’s significant value for broader adoption in clinical education.

## Appendix

1. Strangulated intestinal obstruction refers to intestinal obstruction accompanied by ( ).
  A. Complete obstruction at both ends of the intestinal loop.
  B. Impediment of blood circulation in the intestinal wall.
  C. Perforation and necrosis of the intestinal wall.
  D. Volvulus of the mesentery.
  E. Significant dilatation of the intestinal lumen.
2. A patient with abdominal pain, nausea and vomiting, hyperactive bowel sounds, and cessation of defecation and flatulence should be considered as ( ).
  A. Acute gastroenteritis.
  B. Acute appendicitis.
  C. Acute cholecystitis.
  D. Acute intestinal obstruction.
  E. Acute pancreatitis.
3. The most important aspect in the diagnosis of intestinal obstruction is to determine ( ).
  A. The cause of obstruction.
  B. Whether there is an impediment to blood circulation in the intestinal wall.
  C. The level of intestinal obstruction (high or low).
  D. The degree of obstruction.
  E. The speed of obstruction occurrence.
4. Clinical signs of mechanical intestinal obstruction with intestinal narrowing are manifested as ( ).
  A. Severe colicky abdominal pain, hyperactive bowel sounds.
  B. Significant and symmetric abdominal distention.
  C. Bilious vomiting and gastrointestinal decompression fluid.
  D. Abdominal X-ray showing isolated and prominent dilated intestinal loops that change position over time.
  E. Obvious signs of peritoneal irritation.
5. The most characteristic manifestation of sigmoid volvulus is ( ).
  A. Common in children under 2 years old.
  B. Frequent alternation of diarrhea and constipation.
  C. Abdominal X-ray film showing a horseshoe-shaped, large double-cavity air-filled intestinal loop.
  D. Low-pressure enema often灌注1000ml, but unable to be expelled.
  E. Barium enema showing obstruction at the site of torsion, presenting as a “cup mouth” shape.
6. The three major typical clinical manifestations of infantile intussusception are ( ).
  A. Abdominal pain, bloody stool, abdominal mass.
  B. Abdominal pain, crying, abdominal mass.
  C. Abdominal pain, vomiting, abdominal mass.
  D. Abdominal pain, bloody stool, vomiting.
  E. Abdominal pain, bloody stool, vomiting.
7. The most meaningful laboratory test item in distinguishing simple intestinal obstruction from strangulated intestinal obstruction is ( ).
  A. Blood gas analysis.
  B. Hemoglobin measurement.
  C. White blood cell count.
  D. Routine urine test.
  E. Occult blood test in vomit.
8. If bloody fluid with a foul smell is aspirated during abdominal paracentesis, the most likely diagnosis is ( ).
  A. Acute edematous pancreatitis.
  B. Acute suppurative cholecystitis.
  C. Retroperitoneal hematoma.
  D. Complete strangulated intestinal obstruction.
  E. Rupture of ectopic pregnancy.
9. The most reliable evidence for diagnosing low intestinal obstruction is ( ).
  A. Abdominal plain film showing multiple step-like air-fluid levels in the small intestine.
  B. Auscultation of gurgling sounds around the umbilicus.
  C. Paroxysmal abdominal distention with pain.
  D. Significant alleviation of obstruction symptoms after placing a gastrointestinal decompression tube.
  E. Frequent vomiting with large amounts of vomit.
10. The most important characteristic of abdominal pain in simple mechanical intestinal obstruction is ( ).
  A. Persistent dull pain.
  B. Persistent colicky pain.
  C. Intermittent dull pain.
  D. Persistent abdominal pain.
  E. Colicky pain.
11. A 70-year-old male with intermittent abdominal pain accompanied by abdominal distention, cessation of flatulence and defecation for 3 days. There have been similar episodes in the past, but they were milder. On examination: P 100 beats/min, BP 110/70 mmHg, tense abdominal muscles, significant tenderness with positive rebound tenderness, positive shifting dullness, the most likely diagnosis is ( ).
  A. Paralytic intestinal obstruction.
  B. Incomplete adhesive intestinal obstruction.
  C. Strangulated intestinal obstruction.
  D. Complete high intestinal obstruction.
  E. Simple mechanical obstruction.
12. A 39-year-old female with abdominal pain for 12 h, persistent with paroxysmal exacerbations, accompanied by vomiting, no anal defecation or flatulence. On examination: tense abdominal muscles with tenderness and rebound tenderness. Abdominal paracentesis yielded bloody fluid with a foul smell. The most likely diagnosis for this patient is ( ).
  A. Strangulated intestinal obstruction.
  B. Perforated gastroduodenal ulcer.
  C. Perforated appendicitis.
  D. Tuberculous peritonitis.
  E. Severe acute pancreatitis.
13. A 33-year-old male. Five days after adhesion relaxation surgery for adhesive intestinal obstruction, there has been no anal flatulence, the patient has abdominal distention and feels weak. On examination: normal body temperature, no significant tenderness in the abdomen, no bowel sounds on auscultation. White blood cell count 8 × 10^9/L, small air-fluid levels visible on abdominal radiography. The most likely diagnosis is ( ).
  A. Adhesive intestinal obstruction.
  B. Abdominal hemorrhage with infection.
  C. Intestinal perforation with peritonitis.
  D. Respiratory alkalosis.
  E. Postoperative hypokalemia.
14. A 26-year-old male. Abdominal pain for 2 h after a heavy meal and vigorous activity, persistent pain with paroxysmal exacerbations, periumbilical and lumbosacral pain, frequent vomiting without relief after vomiting. Tense abdominal muscles, tenderness and rebound tenderness around the umbilicus, hyperactive bowel sounds, and auscultation of gurgling sounds. The most likely diagnosis is ( ).
  A. Gastric volvulus.
  B. Acute hemorrhagic necrotizing enteritis.
  C. Small bowel volvulus.
  D. Mesenteric vascular thrombosis.
  E. Intussusception.
15. A 26-year-old male. Abdominal pain for 2 h after a heavy meal and vigorous activity, persistent pain with paroxysmal exacerbations, periumbilical and lumbosacral pain, frequent vomiting without relief after vomiting. Tense abdominal muscles, tenderness and rebound tenderness around the umbilicus, hyperactive bowel sounds, and auscultation of gurgling sounds. The patient needs intravenous infusion of ( ).
  A. Plasma.
  B. Blood substitute.
  C. Whole blood.
  D. Isotonic saline.
  E. Amino acid compound.
16. A 72-year-old male, with paroxysmal abdominal pain for 2 years, feels “gas lumps” moving in the abdomen, initially with increased bowel movements, and in the last 4 months, abdominal distention and constipation, no anal flatulence or defecation for the last 3 days, vomit with fecal odor, and persistent weakness and low-grade fever. Considering the medical history, the intestinal obstruction is ( ).
  A. High incomplete obstruction.
  B. Ischemic intestinal obstruction.
  C. High complete obstruction.
  D. Low incomplete obstruction.
  E. Low complete obstruction.
17. A 72-year-old male, with paroxysmal abdominal pain for 2 years, feels “gas lumps” moving in the abdomen, initially with increased bowel movements, and in the last 4 months, abdominal distention and constipation, no anal flatulence or defecation for the last 3 days, vomit with fecal odor, and persistent weakness and low-grade fever. The most likely cause of obstruction for this patient is ( ).
  A. Tumor.
  B. Mesenteric thrombosis.
  C. Fecal impaction.
  D. Inflammatory stenosis.
  E. Adhesion band.
18. A 72-year-old male, with paroxysmal abdominal pain for 2 years, feels “gas lumps” moving in the abdomen, initially with increased bowel movements, and in the last 4 months, abdominal distention and constipation, no anal flatulence or defecation for the last 3 days, vomit with fecal odor, and persistent weakness and low-grade fever. The contraindicated examination is ( ).
  A. Abdominal ultrasound (B-mode).
  B. Barium meal follow-through.
  C. Abdominal X-ray film.
  D. Colonoscopy.
  E. Abdominal CT.
19. A 15-month-old boy. Sudden crying for 3 h, paroxysmal episodes with normal behavior between episodes, pale complexion during episodes accompanied by vomiting, vomit is milk, and stool is currant jelly-like. During an episode, the most likely abdominal sign on examination is ( ).
  A. Generalized abdominal distention, visible intestinal patterns.
  B. Disappearance of liver dullness.
  C. Weak or absent bowel sounds.
  D. Sausage-shaped mass palpable in the right abdomen.
  E. Generalized abdominal.
20. A 15-month-old boy. Crying suddenly for 3 h, with paroxysmal episodes; appears normal between episodes, but during episodes has a pale complexion accompanied by vomiting, vomits milk that was consumed, and has currant jelly-like stools. The conventional first-line treatment method is ().
  A. Emergency laparotomy.
  B. Low-pressure air enema.
  C. Intravenous administration of antibiotics.
  D. Gastrointestinal decompression.
  E. Sedation and analgesic medication.

**Table 1:** Learning satisfaction score

	5	10	15	20	25
Course content					
Faculty					
Teaching methods					
Course organization					

**Table 2:** Compliance assessment

	5	10	15	20	25
Pre-class self-study and post-class review					
Participation in classroom teaching					
Making learning plans					
Seeking feedback and help					

## Data Availability

The datasets used and/or analysed during the current study available from the corresponding author on reasonable request.

## Abbreviations

ChatGPT: Chat Generative Pre-trained Transformer
USMLE: United States Medical Licensing Examination
ANOVA: Analysis of variance
